# Cancer and Its Complications

**Published:** 1889-12

**Authors:** 


*Cancer and its Complications.* By CHARLES E. JENNINGS, F.R.C.S. Pp. 154. London: BAILLIÈRE & CO. 1889.

It is not easy to understand why this work was written. It contains nothing that is new, either in pathology or treatment, and much that is controversial or erroneous, or antiquated in both. The author in the preface gives, as reasons for the writing of the book, the elucidation of the following points:

1. Cancer is a local disease.

2. There are several varieties of cancer, distinguishable by their microscopic character; but the degree of malignancy of a cancerous growth depends not merely upon its structure, but upon its anatomical site.

4. The occurrence of cancer may often be prevented; and complete extirpation may be considered curative.

5. Further investigation of the action of certain specific drugs—among other things—is still needed. Evidence which has been advanced in favour of some mineral and vegetable substances cannot be disregarded.

As an introduction to these theses, we are treated to a description of the varieties of cancer. For instance:

"MELANOTIC.

| | | |
|---|---|---|
| *Latin equivalent* | ... ... | Carcinoma melanoticum. |
| *French* ,, | ... ... | Mélanose. |
| *German* ,, | ... ... | Melanotischer krebs. |
| *Italian* ,, | ... ... | Cancro melanotico. |

"Melano-carcinomata are much rarer than melano-sarcomata. They are carcinomata in which a deposition of pigment has taken place, giving the tumour a black or grey appearance." That is all. We have much of equivalent naming, if we have little of the thing itself. And that little we would have been better without.

As to the theses themselves, surely never were important scientific subjects approached in a more superficial and even flippant manner. The latter epithet is particularly applicable to the author's method of dealing with the arguments and facts of one who is recognised over the civilised world as a calm and close reasoner, and one not devoid of authority on the subject with which the book deals: we refer to Sir James Paget.

Here is the latest exposition of what we know about cancer of the kidney, in a work specially devoted to cancer:

"The kidney may be attacked by either sarcoma or carcinoma, the former variety often occurring in the young as well as in the old, the latter attacking those past middle life. The number of cases operated upon are not sufficient to justify any strong assertions as to the advisability or otherwise of ever performing this operation; and the results naturally vary, not merely with the character of the disease, but with the skill of the operator.

"Extirpation of a kidney can, of course, only be recommended where the remaining kidney is healthy. The smaller the size of the growth, the greater will be the probability of success. The advantages claimed for the lumbar operation are the better opportunities for drainage and an extraperitoneal operation. By the abdominal incision, a larger tumour may be removed, and a manual exploration of the interior of the abdominal cavity effected.

"Sir Spencer Wells points out that, after excision of the kidney by the abdominal method, the posterior layer of the peritoneum, which was divided to reach the organ, should be reunited, and the smooth peritoneal surfaces brought into apposition."

Our readers will probably agree with us that the best that can be said about the above is, that it closely resembles the sort of answer one would expect from a doubtful candidate for qualification who was asked to write down all he knew about cancer of the kidney. And this is a fair sample of the whole book, even as regards the somewhat forced introduction of Sir Spencer Wells' name.

